# Characterization of two acetyltransferase genes in the pyripyropene biosynthetic gene cluster from *Penicillium coprobium*

**DOI:** 10.1080/13102818.2014.960140

**Published:** 2014-10-31

**Authors:** Jie Hu, Ayako Furutani, Kentaro Yamamoto, Kazuhiko Oyama, Masaaki Mitomi, Hiroyuki Anzai

**Affiliations:** ^a^ State Key Laboratory of Natural and Biomimetic Drugs, Peking University, Beijing, China; ^b^ Gene Research Center, Ibaraki University, Ibaraki, Japan; ^c^ Agricultural & Veterinary Research Laboratories, Meiji Seika Pharma Co., Ltd., Kanagawa, Japan

**Keywords:** acetyltransferase, *Penicillium coprobium*, pyripyropene, biosynthesis, transformants

## Abstract

Pyripyropenes potently and selectively inhibit acyl-CoA:cholesterol acyltransferase 2 (ACAT-2). Among multiple isomers of pyripyropene (A to R), pyripyropene A (PyA) has insecticidal properties in addition to its growth inhibition properties against human umbilical vein endothelial cells. Based on the predicted biosynthetic gene cluster of pyripyropene A, two genes (*ppb8* and *ppb9*) encoding two acetyltransferases (ATs) were separately isolated and introduced into the model fungus *Aspergillus oryzae*, using the protoplast–polyethylene glycol method. The bioconversion of certain predicted intermediates in the transformants revealed the manner by which acetylation occurred in the biosynthetic pathway by the products expressed by these two genes (AT-1 and AT-2). The acetylated products detected by high-performance liquid chromatography (HPLC) in the extracts from AT-1 and AT-2 transformant clones were not present in the extract from the transformant clone with an empty vector. The HLPC charts of each bioconversion study exhibited high peaks at 12, 10.5 and 9 min, respectively. Further ultraviolet absorption and mass spectrometry analyses identified the products as PyE, PyO and PyA, respectively. AT-1 acetylated the C-1 of deacetyl-pyripyropene E (deAc-PyE), while AT-2 played an active role in acetylating the C-11 of 11-deAc-PyO and C-7 of deAc-PyA at two different steps of the biosynthetic pathway.

## Introduction

Pyripyropenes are well-known as acyl-CoA:cholesterol acyltransferase inhibitors. Pyripyropene A (PyA) has been shown to be produced by *Penicillium coprobium* PF1169 [1] and to exhibit insecticidal properties.[2] Among known pyripyropene isomers, pyripyropene A, B and D from the marine-derived fungus *Aspergillus* sp. GF5 are reported to have selective growth-inhibiting properties against human umbilical vein endothelial cells (HUVECs).[3] Based on the findings with a newly developed cell-based assay using ACAT-1- or ACAT-2-expressing Chinese hamster ovary (CHO) cells, PyA was identified as a potent and selective inhibitor of ACAT-2. The in vivo efficacy of PyA in atherosclerosis has been also demonstrated in atherogenic mice.[4] PyA is also reported to exhibit insecticidal properties against *Helicoverpa zea* larvae in agricultural field conditions [2] as well as very strong ACAT-2 inhibitory activity [5] in health science experiments. Therefore, PyA would be a promising drug both in health science and agriculture.

Previous studies suggest that the biosynthesis of pyripyropenes involves polyketide synthase (PKS), which utilizes nicotinic acid-derived coenzyme A (CoA), nicotinyl-CoA, as a starter unit to form the pyrone moiety. The subsequent attachment of a farnesyl group by prenyltransferase (PT) is followed by epoxidation and cyclization to give the basic core structure. Both hydroxylation and acetylation-propionylation are then required to produce various pyripyropenes.[6] Previous biochemical studies partially delineated the pyripyropene A biosynthetic pathway.[7] The biosynthesis of deAc-PyE (the basic core structure of pyripyropenes) in *Aspergillus fumigatus* FO-1289 was precisely elucidated using a transgenic approach with a heterologous fungus. We previously described the formation of the 13-hydroxy group and 11-deAc-PyO by the actions of two P450 genes.[6] However, the mechanisms responsible for the formation of three acetyl groups currently remain unknown.

We deduced three steps to form the three acetyl groups in the biosynthesis pathway of PyA. Only two, out of the nine genes known to be involved in the biosynthesis of PyA, encoded proteins with predicted acetyltransferase (AT) function. Both potential acetyltransferases were *O*-acetyltransferase type. In the present study, we describe the two acetyltransferase genes involved in the biosynthesis of PyA. Each gene was expressed in the heterologous host fungus *Aspergillus oryzae* HL-1105, and functional analysis was performed by feeding several predicted intermediates of pyripyropenes into the culture medium of each transformant clone using high-performance liquid chromatography (HPLC) and liquid chromatography–mass spectrometry (LC–MS).

## Materials and methods

### Strains and culture conditions

*P*. *coprobium* PF1169 (Meiji Seika Pharma Co., Ltd.) and *A*. *oryzae*, the *sC* (adenosine triphosphate sulphurylase) mutant strain HL-1105 (Hakutsuru Sake Brewing Co., Ltd., Kobe, Japan), were used in this study. *P*. *coprobium* was grown on culture plates containing CMMY medium (corn meal 0.85%, Bacto Agar 0.75%, malt extract 1%, yeast extract 0.2%) for 5 days at 28 °C. *P*. *coprobium* was then inoculated into nutrient broth (NB) liquid media (nutrient broth 0.8%, yeast extract 0.2%, KNO_3_ 0.05%) in 500 mL Erlenmeyer flasks, and cultured at 28 °C for 3 days on a rotary shaker (120 r.p.m). Mycelia were then harvested and prepared for DNA extraction.

In the bioconversion analysis, *A*. *oryzae* HL-1105 for transformation was grown in Czapek–Dox medium (NaNO_2_ 0.3%, KCL 0.2%, KH_2_PO_4_ 0.1%, MgSO_4_·7H_2_O 0.05%, trace elements solution 1 mL L^−1^, glucose 2%, methionine 40 μg mL^−1^). As a negative control, wild-type *A*. *oryzae* HL-1034 was grown in Czapek–Dox medium without methionine. The fungal spores were cultured in YDP (yeast extract 0.5%, dextrin 2%, polypeptone 1%) with 1% maltose.

### Bioinformatics analysis

The full PyA biosynthesis gene cluster for *P*. *coprobium* has been described previously.[6] Protein BLAST and nucleotide BLAST were performed at the National Center for Biotechnology Information (NCBI) website (http://www.ncbi.nlm.nih.gov/), using the predicted amino acid sequences encoded by *ppb8* and *ppb9*, respectively. The trees were constructed using MEGA version 5 [8] after a sequence alignment using ClustalW. The maximum likelihood phylogeny was constructed using MEGA version 5 software with a bootstrap of 500.

### Isolation of genomic DNA from *P*. *coprobium*

Genomic DNA was prepared using the QIAGEN Genomic DNA Kit (QIAGEN K. K, Tokyo, Japan) according to the protocol described previously.[6] Isolated DNA was evaluated for integrity on an 0.8% agarose gel, and concentrations were determined spectrophotometrically.

### Construction of expression plasmids

The expression vector pUSA [9] with an α-amylase promoter was used to express the acetyltransferase 1 (AT-1) gene and AT-2 gene in *A*. *oryzae*. Each gene was amplified by polymerase chain reaction (PCR) with a template of the fosmid clone G7-9 from a genomic library, and then inserted into pUSA at *Kpn*I/*Sma*I (see Figure S1 in the Online Supplemental data). The *sC* gene on the plasmid was used as the selectable marker for fungal transformation as described previously.[6]

### Transformation of *Aspergillus oryzae*

*A*. *oryzae* protoplasts were obtained and transformed according to standard methods.[10] All compressed genes were placed under the α-amylase promoter and plasmids were introduced into the adenosine triphosphate (ATP) sulphurylase mutant *A*. *oryzae* HL-1105 strain, using the protoplast–polyethylene glycol method to give transformants, and were then selected for ATP sulphurylase. An average 10 μg expression plasmid was used for transformation. Each independent transformant was grown, harvested and analyzed. The DNA for each transformant clone was extracted in accordance with the modified protocol.[11] Transformants carrying the respective AT genes were verified by PCR analysis using MightyAmp^TM^ DNA polymerase (Takara Bio. Inc., Japan) with the primers listed in Table 1. The primer pairs 40F and AT R with Kpn were used for the transformant harbouring *ppb8*. The primer pairs 70F and infusion R of toxin were used to verify the transformant harbouring *ppb9*. Reactions were cycled at 98 °C for 2 min and 35 cycles of 98 °C for 10 s, 60 °C for 15 s and 68 °C for 1 min.

**Table 1.** List of the primer pairs used in this study.

Primer	Sequence (from 5′ to 3′)	Reference
AT (AT-1) F with Swa	ATTTAAATGTCGTACATATGCTATG	Full-length genomic DNA of AT-1
AT (AT-1) R with Kpn	GCGGTACCACAACTCAACTCAATAGG	
40 F	GATTTCCTGCTCCTCAGTGC	Confirm transformant AT-1
Infusion F of toxin (AT-2)	CCGAATTCGAGCTCGGTACCTCGCTATTGTCAGTTACACA	Full-length genomic DNA of AT-2
Infusion R of toxin (AT-2)	CTACTACAGATCCCCGGGGAACAATCCCGACACATGAA	
70 F	GTATGCACCATCCGTGGAGT	Confirm transformant AT-2

### Metabolite analysis

To determine the function of each open reading frame, wild type *A*. *oryzae* HL-1034 and the positive transformants of *A*. *oryzae* HL-1105 with plasmids containing AT genes were grown at 30 °C on Czapek–Dox medium for 7 days. One hundred microliters of the fungal spores (10^4^ spores mL^−1^) of each transformant were transferred into a 50 mL flask with 10 mL YDP, which contained 1% maltose, and were fed with the predicted intermediates: 11-deAc-PyO, deAc-PyA and deAc-PyE, separately. The culture was agitated at 25 °C. The bioconversion products of the transformants were processed after 24–96 h using culture samples from the mycelia and broth taken at each time point. All these processes were performed as described previously.[6] The mycelia and broth mixture was treated with acetone and thoroughly mixed. Acetone was removed by a centrifugal concentrator and ethyl acetate was added to extract the total products. Then the ethyl acetate extract was concentrated in vacuum to dryness. Individual extracts were dissolved in 1 mL of methanol and analyzed with LC–MS on a Micromass ZQ (Waters, MA, USA), 2996 Photodiode array (Waters), 2695 Separation module (Waters) and Xterra C18 column (*ϕ* 4.5 mm × 50 mm, 5 μm; Waters). A linear gradient (eluent: acetonitrile-water (v/v) 20% to 100% in 26 min, flow rate 0.8 mL min^−1^ at 40 °C) was used for analysis of bioconversion products. All were detected at 320 nm.

## Results and discussion

To identify all the biosynthetic genes needed to produce PyA, the putative biosynthetic gene cluster that contained both PKS and PT genes was obtained based on the genomic DNA database of *P*. *coprobium* through 454 FLX sequencing.[6] We previously identified a gene cluster that spanned 24 kb with nine genes, including CoA ligase, PKS, cytochrome P450 (P450-1), P450-2, integral membrane protein (IMP), flavin adenine dinucleotide (FAD)-dependent monooxygenase (FMO), prenyltransferase (PT) and acetyltransferase (AT-1 and AT-2). Four R groups were identified for pyripyropenes: C-1, C-11, C-7 and C-13. The inhibition of ACAT-2 from a structure-activity relationship (SAR) study of PyA derivatives showed that: (1) the 13-hydroxy group was necessary; (2) 1,11-dihydroxy position could be modified and the acyl group at the 7-*O*-hydroxyl position was necessary.[12,13]

### Bioinformatics analysis and phylogenetic analysis

We here focused on two genes encoding ATs. Based on the deduced amino acid sequence, the molecular weight of AT-1 (522 amino acids) was calculated to be approximately 57.7 kD and that of AT-2 (434 amino acids) was approximately 49 kD (Table 2). When analyzed by blastx with genomic DNA sequences, AT-1 showed 98% coverage to the acetyltransferase of *Neosartorya fischeri* NRRL 181 and 72% identity, while AT-2 showed 96% coverage to *A*. *fumigatus* A1163 and 75% identity. Regarding the genomic structure of the two AT genes, the AT-2 gene had two introns, whereas the AT-1 gene had none.

**Table 2.** Deduced functions of each open reading frame and their amino-acid sequence coverage and identity with other known proteins are shown (the genes described herein and their functions are identified by grey backgrounds).

Gene	Amino acids (genomic DNA base pairs)	MW (kD)	Protein homologue, origin	Coverage/identity (%)	Proposed functions	Number of exons
*ppb1*	556 (1514)	61.3	4CL, *Neosartorya fischeri*	91/82	CoA ligase	2
*ppb2*	2,447 (7396)	266.1	LovB-like polyketide synthase, *Aspergillus fumigatus*	99/72	Polyketide synthase	2
*ppb3*	509 (1879)	57.3	Cytochrome P450, *Neosartorya fischeri*	88/83	Cytochrome P450 monooxygenase-1	6
*ppb4*	505 (1799)	56.6	Cytochrome P450, *Neosartorya fischeri*	95/75	Cytochrome P450 monooxygenase-2	6
*ppb5*	241 (791)	27.4	Integral membrane protein, *Aspergillus fumigatus*	92/84	Integral membrane protein	2
*ppb6*	464 (1611)	51.4	PaxM, *Aspergillus fumigatus*	84/82	FAD-dependent monooxygenase	4
*ppb7*	317 (1085)	35.4	UbiA-like prenyltransferase, *Aspergillus fumigatus*	89/84	UbiA-like prenyltransferase	3
*ppb8*	522 (1569)	57.7	Acetyltransferase, *Aspergillus fumigatus*	98/70	Acetyltransferase-1	0
*ppb9*	434 (1355)	49	Tri7, *Aspergillus fumigatus*	96/75	Acetyltransferase-2	2

**Figure 1.**
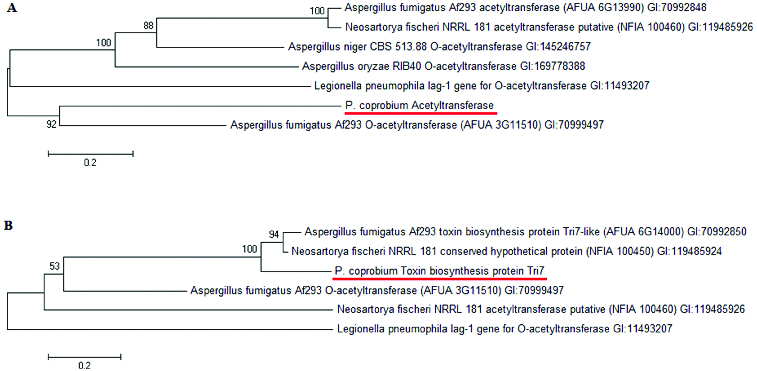
TreePlot showing the homology of AT-1 and AT-2 in *P*. *coprobium* compared to other acetyltransferases from other species and strains (Mega 5.1 software, Invitrogen Co. Ltd.).

The phylogenetic analysis of the acetyltransferases from *Aspergillus*, *Neosartorya* and *Legionella* revealed that the *ppb8* gene encoding AT-1 of *P*. *coprobium* shared strong homology to *A*. *fumigatus* Af293 *O*-acetyltransferase (Figure 1(A)), and the *ppb9* gene encoding AT-2 shared very strong homology to toxin biosynthesis protein Tri7 (Figure 1(B)). That is, the sequence of *P*. *coprobium* acetyltransferase AT-1 was 23205–24773 from GenBank accession no. FW308713. The sequence of *P*. *coprobium* toxin biosynthesis protein Tri7 (AT-2) was 25824–27178 from GenBank accession no. FW308713.

The results of the bioinformatics analyses indicated that the protein products of these two genes may be involved in acyltransferase reactions.

### Generation of AT transformants in the *A. oryzae* host

To characterize the function of both gene products, each protein was expressed in the heterologous fungal host *A*. *oryzae* [14,15] HL-1105 rather than disrupting each gene in the cluster of the producer strain *P*. *coproium* PF1169. This fungal expression system was used successfully to characterize fungal genes functionally, such as the P450 genes, *brlA* [16] and *abaA*.[17] Thus, the function of each enzyme was characterized directly, and novel meroterpenoids were produced by the incorporation of different starter units or intermediates in the pathway.

The earliest candidate single clone was found on the third day. The candidates were selected until day 7, and the average number of selected clones in transformation experiments was approximately 10–15. The candidate single clonal transformants were then transferred to separate dishes and cultured for functional analysis. Before the bioconversion analysis, the transformants harbouring AT genes were confirmed through genomic PCR (see Figure S2 in the Online Supplemental data).

### Bioconversion analysis

The expression of the predicted AT-1 gene (*ppb8*) was carried out in the *A*. *oryzae* host. Three transformant clones were cultured in YDP media supplemented with maltose to induce expression, and deAc-PyE, 11-deAc-PyO and deAc-PyA (final concentration of 20 μg mL^−1^) were fed into the induction culture separately. The transformants were cultured for 48–96 h. A product was detected by HPLC analysis at a retention time (RT) of 12 min, but was not found in the control transformant with an empty vector (Figure 2(A)). Further characterization was performed by LC–MS and the structure of the product was identified. It gave a quasimolecular ion peak at *m*/*z* 452 [M+H]^+^ as PyE, which has already been identified,[18] and its molecular formula was determined to be C_27_H_33_NO_5_. It also showed the same ultraviolet (UV) absorption at 210 and 320 nm as reported previously.[18] The expected PyE was detected in the culture fed with deAc-PyE. Neither PyO, nor PyA was detected in the extract of the culture when fed with 11-deAc-PyO or deAc-PyA. There was no difference among the three transformant clones. These results suggest that the *ppb8* gene encodes a protein with acetyltransferase activity that selectively acted on the 1-hydroxy position of deAc-PyE. Figure 2. HPLC profiles of metabolites. Culture feeding with DeAc-PyE/ AT-1 (A), DeAc-PyA (B) or 11-deAc-PyO (C). Transformants harboured AT-1 (A) or AT-2 (B, C). The transformant that harboured an empty vector was a control in all feeding tests.

**Figure:**
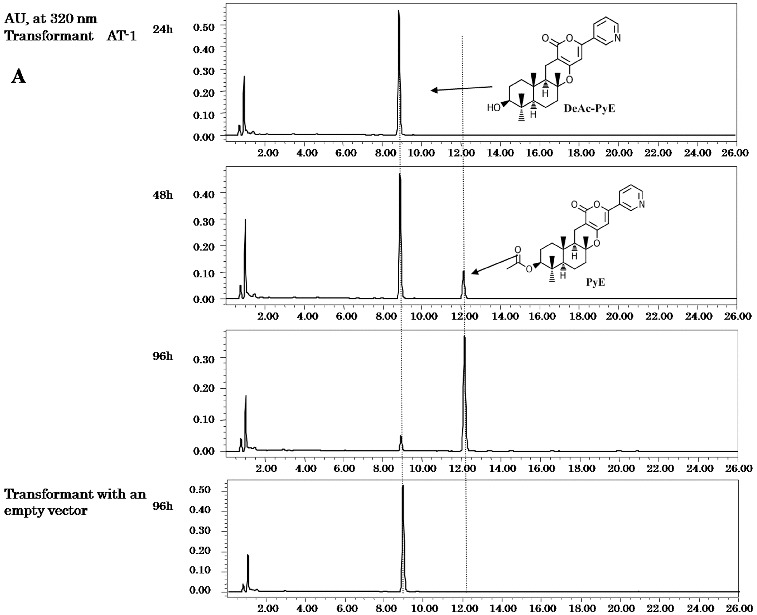

**Figure:**
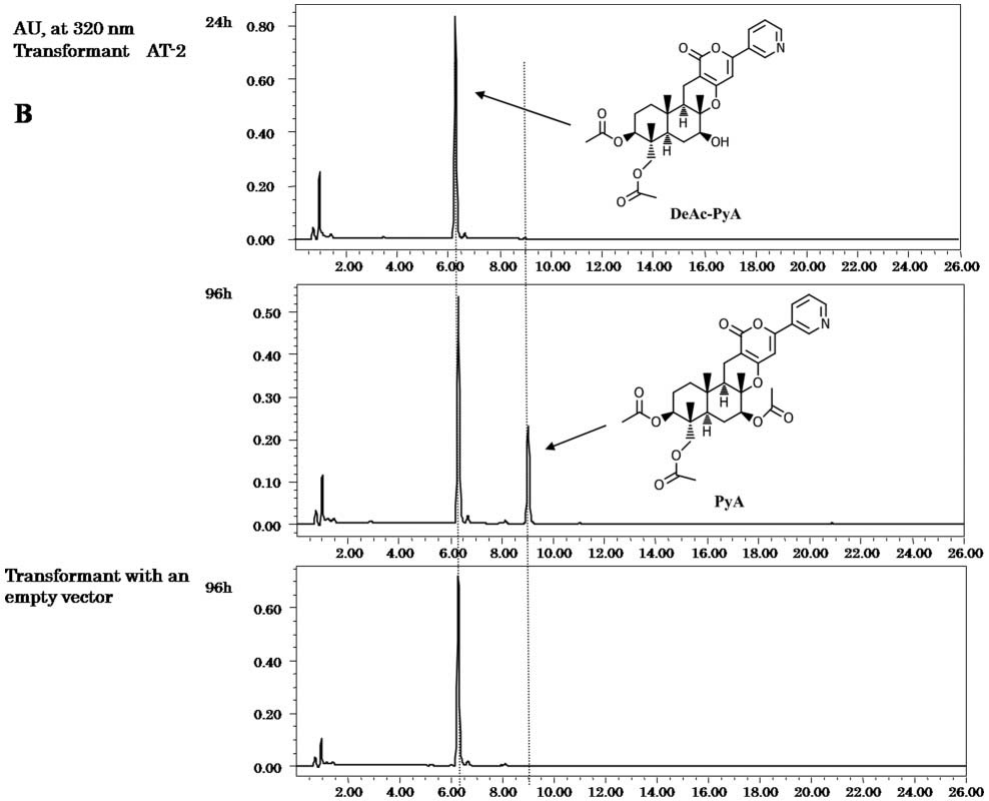

**Figure:**
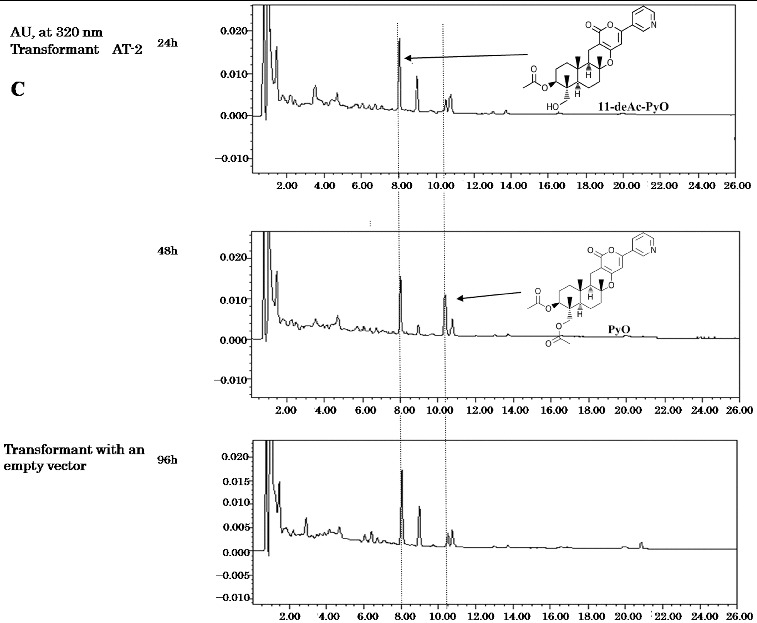


**Figure 3.**
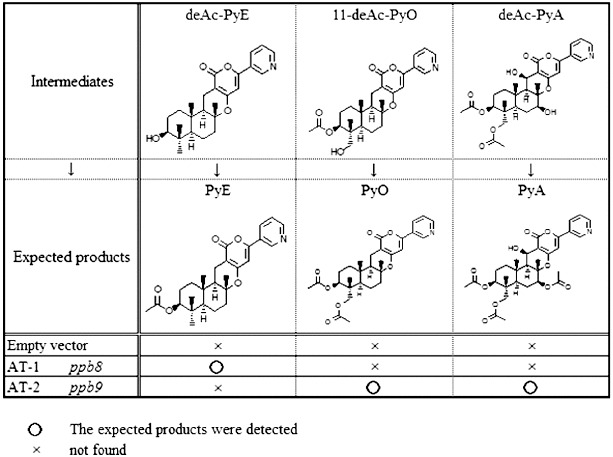
Bioconversion test with predicted intermediates.

The two other expected acetylation steps at C-7 and C-11 should be a result of the activity of one or more other acetyltransferase genes. First, the AT-2 gene (*ppb9*) encoding a predicted toxin biosynthesis protein Tri7 was introduced into the host fungi as described above. Three transformant clones were also fed with deAc-PyE, 11-deAc-PyO and deAc-PyA, respectively. The expected product PyE was not detected in feeding with deAc-PyE (Figure 3). In the extract of the transformants supplied with 11-deAc-PyO, the peak at RT = 10.5 min became clear after 48 h of cultivation (Figure 2(C)). LC–MS analysis gave a quasimolecular ion peak at *m*/*z* 510 [M+H]^+^ as PyO, which has already been identified,[19] and the molecular formula was determined to be C_27_H_35_NO_7_. It also showed the same UV absorption at 231 and 320 nm as reported previously.[19] When the transformants expressing the *ppb9* gene were fed with deAc-PyA, the HLPC chart exhibited a high peak at RT = 9 min from the extract of culture after 96 h (Figure 2(B)). The compound gave a quasimolecular ion peak at *m*/*z* 584 [M+H]^+^ as PyA, which has already been identified,[20] and the molecular formula was determined to be C_31_H_37_NO_10_. It also showed the same UV absorption at 231 and 320 nm as PyA.[20] These three transformant clones also showed no difference in bioconversion analysis. The presented results of the biotransformation analysis indicate that the *ppb9* gene encodes a protein with acetyltransferase activity that is involved in C-7 and C-11 acetylation in two separate steps of the PyA biosynthesis pathway.

**Figure 4.**
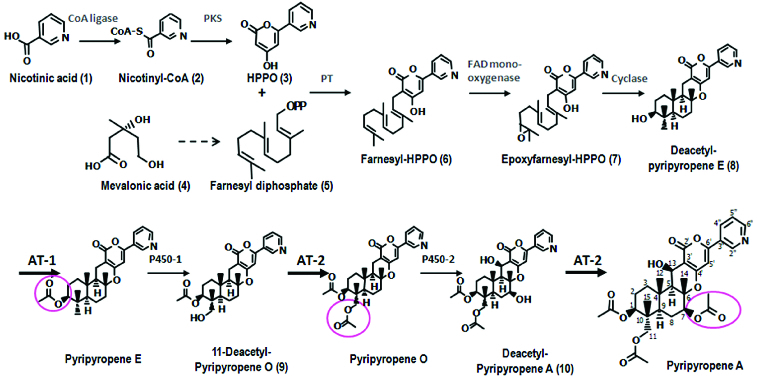
Proposed biosynthetic pathway for pyripyropene A. Steps (1)–(8) were reported previously by Itoh et al.[21] Based on the results of bioconversion, the next steps were deduced to be acetylation by AT-1 and AT-2 and hydroxylation by two P450s.

Including our previous findings, the basic core structure, deAc-PyE, was first acetylated at 1-hydroxy by the expression of AT-1 and PyE was formed (Figure 4). It was subsequently hydroxylated by P450-1 to form 11-deAc-PyO,[6] which was subsequently converted to PyO by the activity of AT-2 at the 11-hydroxy group. By expressing P450-2, the C-13 and C-7 of PyO were hydroxylated to form deAc-PyA.[6] The 7-hydroxy group was then acetylated by the activity of AT-2. Thus, we elucidated the later steps of the fungal meroterpenoid biosynthetic gene cluster for PyA from *P*. *coprobium*, which has important potential activities. Combined with the biosynthetic pathway of the basic core structure reported by Itoh's group,[21] the biosynthesis of PyA was clarified. The isolation and cloning of these enzymes will greatly enhance our understanding of these processes and will allow engineering of novel hybrid molecules for industrial applications.

## Conclusions

Together with previous findings, the results from this study helped to identify the later steps of the fungal meroterpenoid biosynthetic gene cluster for PyA from *P*. *coprobium*. The *ppb8* gene was shown to encode a protein with acetyltransferase activity that selectively acts on the 1-hydroxy position of deAc-PyE, and the *ppb9* gene to encode a protein with acetyltransferase activity that is involved in C-7 and C-11 acetylation in two separate steps of the PyA biosynthesis pathway. The elucidation of the steps involved in the biosynthesis of PyA and the isolation and cloning of these enzymes will allow engineering of novel promising hybrid molecules for industrial applications both in health science and agriculture.

## Supplemental data

Supplemental data for this article can be accessed here.
